# The Protective Effect of a Novel Cross-Linked Hemoglobin-Based Oxygen Carrier on Hypoxia Injury of Acute Mountain Sickness in Rabbits and Goats

**DOI:** 10.3389/fphys.2021.690190

**Published:** 2021-09-27

**Authors:** Jie Zhang, Yue Wu, Xiao-Yong Peng, Qing-Hui Li, Xin-Ming Xiang, Yu Zhu, Qing-Guang Yan, Billy Lau, Feichuen Tzang, Liang-Ming Liu, Tao Li

**Affiliations:** ^1^State Key Laboratory of Trauma, Burns and Combined Injury, Department of Shock and Transfusion, Research Institute of Surgery, Daping Hospital, Army Medical University, Chongqing, China; ^2^New Beta Innovation Limited, Kowloon Bay, Hong Kong, SAR China

**Keywords:** hemoglobin-based oxygen carrier (HBOC), YQ23, acute mountain sickness (AMS), releasing oxygen function, carrying oxygen function

## Abstract

Hypoxia is the major cause of acute altitude hypoxia injury in acute mountain sickness (AMS). YQ23 is a kind of novel bovine-derived, cross-linked hemoglobin-based oxygen carrier (HBOC). It has an excellent capacity for carrying and releasing oxygen. Whether YQ23 has a protective effect on the acute altitude hypoxia injury in AMS is unclear. In investigating this mechanism, the hypobaric chamber rabbit model and plain-to-plateau goat model were used. Furthermore, this study measured the effects of YQ23 on the ability of general behavior, general vital signs, Electrocardiograph (ECG), hemodynamics, vital organ injury markers, and blood gases in hypobaric chamber rabbits and plain-to-plateau goats. Our results showed that the ability of general behavior (general behavioral scores, GBS) (GBS: 18 ± 0.0 vs. 14 ± 0.5, *p* < 0.01) and the general vital signs weakened [Heart rate (HR, beats/min): 253.5 ± 8.7 vs. 301.1 ± 19.8, *p* < 0.01; Respiratory rate (RR, breaths/min): 86.1 ± 5.2 vs. 101.2 ± 7.2, *p* < 0.01] after exposure to plateau environment. YQ23 treatment significantly improved the ability of general behavior (GBS: 15.8 ± 0.5 vs. 14.0 ± 0.5, *p* < 0.01) and general vital signs [HR (beats/min): 237.8 ± 24.6 vs. 301.1 ± 19.8, *p* < 0.01; RR (breaths/min): 86.9 ± 6.6 vs. 101.2 ± 7.2, *p* < 0.01]. The level of blood PaO2 (mmHg) (115.3 ± 4.7 vs. 64.2 ± 5.6, *p* < 0.01) and SaO2(%) (97.7 ± 0.7 vs. 65.8 ± 3.1, *p* < 0.01) sharply decreased after exposure to plateau, YQ23 treatment significantly improved the blood PaO2 (mmHg) (97.6 ± 3.7 vs. 64.2 ± 5.6, *p* < 0.01) and SaO2(%) (82.7 ± 5.2 vs. 65.8 ± 3.1, *p* < 0.01). The cardiac ischemia and injury marker was increased [troponin (TnT, μg/L):0.08 ± 0.01 vs. 0.12 ± 0.02, *p* < 0.01], as well as the renal [blood urea nitrogen (BUN, mmol/L): 6.0 ± 0.7 vs. 7.3 ± 0.5, *p* < 0.01] and liver injury marker [alanine aminotransferase (ALT, U/L): 45.8 ± 3.6 vs. 54.6 ± 4.2, *p* < 0.01] was increased after exposure to a plateau environment. YQ23 treatment markedly alleviated cardiac ischemia [TnT (μg/L):0.10 ± 0.01 vs 0.12 ± 0.02, *p* < 0.01] and mitigated the vital organ injury. Besides, YQ23 exhibited no adverse effects on hemodynamics, myocardial ischemia, and renal injury. In conclusion, YQ23 effectively alleviates acute altitude hypoxia injury of AMS without aside effects.

## Introduction

Acute mountain sickness is serious of hypoxia-induced symptoms such as headache, fatigue, and insomnia for people who were not acclimatized to rapid exposure of 3,500 m above mean sea level (MAMSL) (Jin, 2017). Journalists, soldiers, and medical workers are at high risk of developing acute mountain sickness (AMS) because they engage in intense physical activity immediately after their rush to the plateau with the tasks of disaster relief or military action. Approximately, the incidence of AMS can be as high as 85%, depending on the MAMSL (Bärtsch and Swenson, 2013). Without treatment, AMS may induce vital organ injuries and rapidly progress pulmonary edema and cerebral edema, which is life-threatening.

Hypoxia is the main cause of AMS which decreases the blood oxygen saturation and utilization, and further induces vital organ damage (Mehta et al., 2008; Barker et al., 2016). Thus, oxygen inhalation is an effective way to prevent and treat mild AMS. However, due to the weight and volume of oxygen tanks, it is very difficult to carry on, especially for soldiers and medical workers with military or disaster relief tasks. Besides, the effect of oxygen inhalation is short. Other preventive measures for severe AMS include *Rhodiola rosea* (RhRo), acetazolamide, and dexamethasone (Tang et al., 2014; Ou et al., 2020; Toussaint et al., 2020). However, these measures are unable to improve tissue hypoxia fundamentally and are unsuitable for emergencies since they take a long time to take effect.

YQ23 is a novel bovine-derived, stabilized, cross-linked hemoglobin-based oxygen carrier (HBOC). Our previous study has demonstrated the protective effects of YQ23 on hemorrhagic shock in pigs and rats and sepsis in rats without aside effects, showing excellent oxygen-carrying and releasing capacity (Li et al., 2017; Kuang et al., 2019). Thus, we hypothesized that YQ23 has a potential protective effect on acute hypoxia injury in AMS. To confirm the hypothesis, the rabbit model was placed in a hypobaric chamber and the goat model was flown from Chongqing to Lhasa to mimic the AMS. The beneficial effect of YQ23 on acute hypoxia symptoms of AMS was investigated. The ability of general behavior, basic vital signs, Electrocardiograph (ECG), hemodynamics, vital organ function, and blood gases were measured.

### Animals and Methods

This study involved 136 male and female rabbits (12–14 weeks, 2.2–2.5 kg) and 48 goats (14–18 months, 45–50 kg) were purchased from the Animal Center of Research Institute of Surgery, Third Military Medical University (Army Medical University) and used in the present study. The present study conformed to the principles of the “Guide for the Care and Use of Laboratory Animals” (Eighth Edition, 2011, National Academies Press, Washington DC) and was approved by the Research Council and Animal Care and Use Committee of the Research Institute of Surgery [Daping Hospital, Third Military Medical University (Army Medical University), Chongqing, P. R. China].

### Preparation of YQ23 and *Rhodiola rosea*

YQ23 products were obtained from New B Innovation Limited, Hong Kong. The purity of YQ23 is above 99.2% with undetectable/low levels of dimeric hemoglobin, met-hemoglobin, phospholipid, and DNA or protein impurities. The osmolality and viscosity (at 37°C) were >250 mOsm/kg and 0.9 centipoises, respectively. The concentration of the YQ23 product was 6.2 g/dL and its pH range was 7.2–7.8. Based on our previous study, two doses of YQ23 (0.3 or 0.5 g/kg) were used in the present study. *R. rosea* was purchased from Tonghua Yusheng Pharmaceutical Co. Ltd, China.

### Experiment Protocol

#### Part 1: The Protective Effect of YQ23 on Acute Hypoxia Injury of Acute Mountain Sickness (AMS) in Hypobaric Chamber Rabbits

At the beginning of the experiment, 136 rabbits were divided into five groups randomly, 8 in the plain control group, 32 in hypobaric chamber groups, 32 in YQ23-0.5 g/kg groups, 32 in YQ23-0.3 g/kg groups, and 32 in RhRo groups. The rabbits in the YQ23-0.3 g/kg and YQ23-0.5 g/kg groups were treated with YQ23 at doses of 0.3 g/kg or 0.5 g/kg respectively. The rabbits in RhRo groups were treated with RhRo as the treatment control group. The rabbits in the hypobaric chamber groups were treated with the same volume of Salinger liquid (Sichuan Kelun Pharmaceutical Co., Ltd, Sichuan, China). The rabbits in the YQ23-0.3 g/kg, YQ23-0.5 g/kg group, RhRo group, and hypobaric chamber group were divided into four subgroups according to the time points after treatment (1, 4, 12, and 24 h).

**FIGURE 1:**
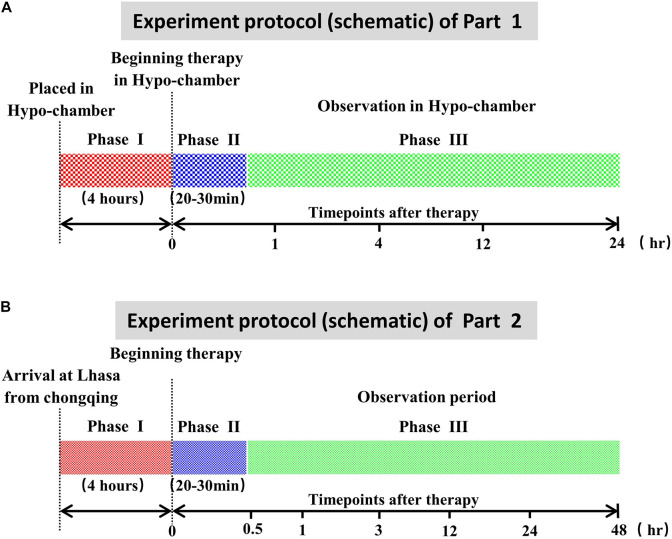
Experiment protocol (schematic). **(A)** Experiment protocol of part 1. Phase I: the acute maintain sickness model stage made in the hypobaric chamber. Phase II: the treatment period with YQ23 or *Rhodiola rosea* (RhRo). Phase III: the parameter measurement at different time points. **(B)** Experiment protocol of part 2. Phase I: the acute maintain sickness model stage. Phase II: the treatment period with YQ23 or *R. rosea* (RhRo). Phase III: the parameter measurement at different time points.

The whole experimental process of this part is shown in Figure 1A. Except for the plain control groups, the rabbits in hypobaric chamber groups, YQ23-0.5 g/kg groups, YQ23-0.3 g/kg groups, and RhRo groups were placed in the hypobaric chamber (Guizhou Fenglei Aviation Ordnance Co., Ltd, Guizhou, China), in which the air pressure was decreased sharply to mimic the plateau environment at 5,000 MAMSL. After 4 h, the rabbits of each group were fixed on the operating table without anesthetization in the hypobaric chamber. After local anesthesia with procaine hydrochloride subcutaneous injection (Huiyinbi Group Jianxi Dongya Pharmaceutical Co. Ltd, China), catheters were inserted into the femoral vein for administration of YQ23 at the dose of 0.5 g/kg, 0.3 g/kg, or RhRo (1 mg/kg) and were inserted into the heart *via* carotid artery for hemodynamics measurement and blood samples. At each time point (1, 4, 12, and 24 h) after treatment, parameters containing heart rate (HR), respiratory rate (RR), general behavioral scores (GBS), and Electrocardiograph (ECG) were measured. Furthermore, 5 ml arterial blood samples were obtained for organ function measurement (3 ml), hemorheology indexes (1 ml), and blood gases indexes (1 ml). Then the rabbit was euthanized for the brain and lung tissues for measurement of brain and lung edema.

#### Part 2: The Protective Effect of YQ23 on Acute Hypoxia Injury of Acute Mountain Sickness (AMS) in Goats in the Plateau (Lhasa)

The whole experimental process of this part is shown in Figure 1B. Before the goats were transported to the Lhasa (3,600 MAMSL) from Chongqing (100 MAMSL), the detaining needle was inserted into the femoral artery for a blood sample and blood pressure. Exactly 3 ml of arterial blood samples were obtained for organ function and hemorheology measurement. Exactly 1 ml of arterial blood samples were obtained for blood gases *via* the ear arteries. Then the same volume of sodium chloride solution was administrated. The goats were transported to Lhasa by airplane, with free eating and drinking in their cages.

Four hours after arrival, catheters were inserted into the ear vein for administration of YQ23 (0.5 g/kg) and RhRo (1 mg/kg). At the time point of 0.5, 1, 3, 12, 24, and 48 h after YQ23 and RhRo treatment, the arterial blood samples were obtained for measuring parameters. At the 48-h time point, the goats were euthanized for brain tissue and lung tissue.

### The Measurement of Parameters

#### The Ability of General Behavior

Given that headache, fatigue, and emesis are the earlier symptoms of AMS, the GBS were used to reflect the symptoms of AMS, including feeding behavior, muscle tone, olfactory stimuli, and so on. The ability of general behavior was measured by scoring quantitative behavioral indicators, including feeding behavior, righting score, muscle tone, motor ability, and so on. Briefly, for feeding behavior, the scores of no food intaking, a little food intaking, and large food intaking were one, two, or three, respectively. For righting scores, we changed the position of the rabbit from supine position to prone position, and the score was determined by the time it returned to the position. The score of the rabbit was one for no recovery, two for recovery after 3 s, and three for immediate recovery. The other detailed information is seen in Table 1.

**TABLE 1:** General behavioral score.

Index	Degrees	Scores	Notes
Feeding behavior	No food intake	1	The score is determined by amount of food intaking in 10 min for three independent repeated experiments
	Food intake was less than 2 g	2	
	Food intake was more than 2g	3	
Righting score	No turning Turning 3 s later Immediate turning	1 2 3	The position of the rabbit is changed from supine position to prone position, and the score is determined by the time it returns to the original position for three independent repeated experiments.
Muscle tone	No resistance The rabbits resisted less than three times during the hind leg extension The rabbits resisted more than three times during the hind leg extension	1 2 3	The rabbit’s hind legs are stretched to the maximum extent, and the score is determined by the times of resistance for three independent repeated experiments by one person.
Motor ability	Immobility The movement distance was less than 1 m The movement distance was more than 1 m	1 2 3	The rabbits were placed on a table and observe the activity within 1 min. The score is determined by the distance it moves for three independent repeated experiments.
Olfactory stimuli	No avoiding Avoiding 3 s later Immediate avoiding	1 2 3	Put the alcohol cotton on the rabbit’s nose, and the score is determined by the time of avoidance for three independent repeated experiments.
Grasping reaction	No reaction Reaction 3 s later Immediate reaction	1 2 3	Grab the rabbit and lift it. The score is determined by the time they struggle

#### Heart Rate (HR) and Respiratory Rate (RR)

Respiratory rate and HR were used to reflect the systemic state of rabbits and goats and were measured by counting per minute.

#### Electrocardiograph (ECG)

For the measurement of ECG, the hair was primarily removed from the joints of the left forelimb, the right forelimb, the right ankle, and the left ankle. Afterward, the red, yellow, black, and green lead lines were attached onto these four places in turn. A portable electrocardiograph (CM1200B, COMEN, Shenzhen, China) was used to monitor the electrocardiograph at every timepoint. The electrocardiograph analysis was done by a cardiologist. The sinus arrhythmia, ST-segment depression, and T-wave change were used to reflect the cardiac ischemia. Briefly, the change of T-wave or the distance of ST-segment moving down more than 0.05 mv suggested myocardial hypoxia, while QRS-wave less than 0.12 s suggested sinus arrhythmia.

#### Blood Gases

At every time point, 1 ml of arterial blood was obtained by heparinized and hermetic injector *via* femoral artery cannula. A blood-gas analyzer (ABL90FLEX, Radiometer, Denmark) was used to measure the PaO_2_, SaO_2_, PCO_2_, PH, HCO_3_^–^, bases excess (BE), and lactic acid in the blood.

#### Vital Organ Function

At every time point, 3 ml of arterial blood was obtained *via* femoral artery cannula, which then was subjected to centrifugation at 3,500 revolutions per minute (rpm) at room temperature. The serum supernatant in the upper level was analyzed by a biochemical analyzer (DX800, Beckman Coulter, 250 S. Kraemer Boulevard Brea, CA, United States). The damage degree of the liver was reflected by the level of glutamic-pyruvic transaminase (ALT) and glutamic-oxalacetic transaminase (AST) in blood. The kidney injury was reflected by urea and creatinine (CREA) and the cardiac injury was reflected by troponin T (TNT) in blood.

#### Brain and Lung Edema

The water content of the brain and lung were determined by the formula: water content = (wet weight-dry weight)/wet weight.

### Statistical Analysis

The GBS, RR, HR, MAP, hemodynamics, blood gas parameters, hemorheology were presented as the *M* ± *SD* of n observations (*n* = 8). The statistical differences among groups were analyzed using two-factor variance analysis, followed by the *post hoc* Tukey test (SPSS v15, SPSS Inc, Chicago, IL, United States) for multiple comparisons between two groups. All data underwent the Kolmogorov–Smirnov normality test and the Bartlett homoscedasticity test. *P* < 0.05 was considered significant.

## Results

### Part 1: The Protective Effect of YQ23 on Acute Hypoxia Injuries of Acute Mountain Sickness (AMS) in Hypobaric Chamber Rabbits

To investigate the potential benefits of YQ23 on acute hypoxia injuries of AMS, a hypobaric chamber rabbit model was used in this part to mimic the AMS. The model was made by placing rabbits in the hypobaric chamber, in which the air pressure decreased sharply to mimic the plateau environment at 5,000 MAMSL within 30 min. Then the protective effect of YQ23 was observed.

#### General Behavioral Scores

**FIGURE 2:**
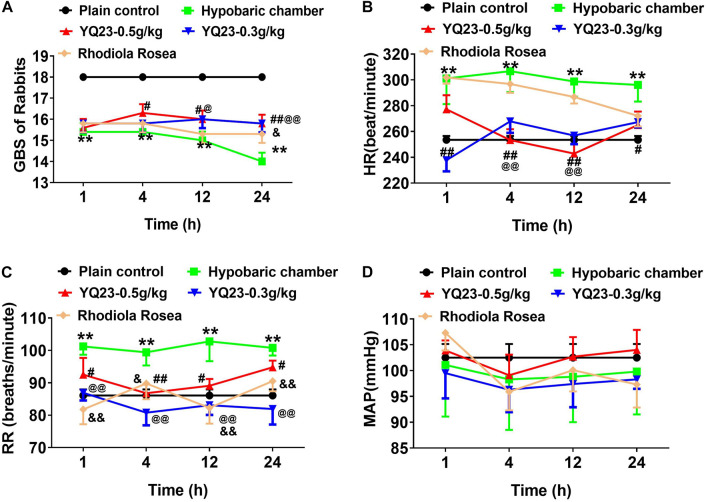
The effect of YQ23 on general behavioral scores (GBS), heart rate (HR), respiratory rate (RR) and mean arterial pressure (MAP) in hypobaric chamber rabbits. **(A)** The effect of YQ23 on GBS; **(B,C)** the effect of YQ23 on HR and RR; **(D)** the effect of YQ23 on MAP. The data are the mean ± SD of n experiments (*n* = 8). **p < 0.01 the hypobaric chamber groups vs. the plain control; ^#^*p* < 0.05, ^##^*p* < 0.01 the YQ23-0.5 g/kg groups vs. the hypobaric chamber groups; ^@^*p* < 0.05, ^@@^*p* < 0.01 the YQ23-0.3 g/kg groups vs. the hypobaric chamber groups; ^&^*p* < 0.05, ^&&^*p* < 0.01 the *R. rosea* groups vs. the hypobaric chamber groups.

The results showed that the GBS significantly reduced after exposure to a hypobaric chamber environment (18.0 ± 0.0 vs. 14.0 ± 0.5, *p* < 0.01) (Figure 2A). With the prolonged exposure time, the GBS continued to decrease (Figure 2A). YQ23 treatment improved the GBS at 4 h (15.8 ± 0.5 vs. 14.0 ± 0.5, *p* < 0.01) and continued for 24 h. *Rhodiola rosea* increased GBS at 24 h (Figure 2A), suggesting YQ23 takes effect earlier than RhRo.

#### Heart Rate, Respiratory Rate, and Mean Arterial Pressure

The HR (253.5 ± 8.7 vs. 301.1 ± 19.8) and RR (86.1 ± 5.2 vs. 101.2 ± 7.2) increased significantly in hypobaric chamber rabbits (Figures 2B,C). YQ23 treatment effectively abrogated the increase of HR (237.8 ± 24.6 vs. 301.1 ± 19.8, *p* < 0.01) and RR (86.9 ± 6.6 vs. 101.2 ± 7.2, *p* < 0.01), while RhRo treatment only decreased the RR but failed to decrease the HR (Figures 2B,C). The mean arterial pressure did not significantly change when exposed to the hypobaric chamber environment (Figure 2D). Notably, YQ23 treatment also did not alter the MAP, suggesting a free vasoconstriction side-effect (Belcher et al., 2014; Figure 2D).

#### Blood Gases

The results showed the levels of PaO_2_ (115.3 ± 4.7 vs. 64.2 ± 5.6, *p* < 0.01) and SaO_2_ (97.7 ± 0.7 vs. 65.8 ± 3.1, *p* < 0.01) decreased sharply in hypobaric chamber rabbits. YQ23 treatment increased the level of PaO_2_ and SaO_2_, and the beneficial effects were maintained for 24 h (Table 2), showing excellent ability to carry and release oxygen. However, RhRo failed to increase the level of PaO_2_ (97.6 ± 3.7 vs. 64.2 ± 5.6, *p* < 0.01) and SaO_2_ (82.7 ± 5.2 vs. 65.8 ± 3.1, *p* < 0.01). Also, the level of blood lactic acid increased after exposure to the hypobaric chamber (9.3 ± 1.9 vs. 11.6 ± 2.1, *p* < 0.05), YQ23 treatment abrogated the increase of blood lactic acid (9.5 ± 1.0 vs. 11.6 ± 2.1, *p* < 0.05) (Table 2). The PCO_2_, blood PH, BE, and HCO_3_^–^ did not change significantly within 24 h.

**TABLE 2:** The effect of YQ23 on blood gases in hypobaric chamber rabbits.

Treatment time	Groups				
	Plain control	Hypobaric chamber	YQ23 0.5 g/kg	YQ23 0.3 g/kg	*Rhodiola rosea*
**PaO_2_ (mmHg)**					
1 h	115.3 ± 4.7	64.2 ± 5.6**	93.2 ± 4.2##	97.6 ± 3.7##	72.5 ± 4.5
4 h		62.9 ± 3.8**	97.5 ± 4.6##	96.2 ± 4.2##	70.0 ± 2.7^#^
12 h		66.3 ± 6.0**	93.3 ± 4.2##	96.8 ± 6.3##	72.6 ± 4.4
24 h		67.2 ± 4.9**	92.1 ± 5.4##	93.6 ± 3.8##	73.7 ± 3.9
**SaO_2_(%)**					
1 h	97.7 ± 0.7	65.8 ± 3.1**	83.5 ± 5.2##	82.7 ± 5.2##	69.1 ± 6.2
4 h		66.4 ± 5.8**	85.4 ± 2.1##	83.8 ± 5.2##	66.2 ± 2.4
12 h		68.4 ± 7.3**	86.5 ± 4.5##	82.0 ± 4.6##	67.1 ± 3.9
24 h		66.8 ± 3.7**	84.6 ± 5.4##	79.8 ± 5.4##	67.6 ± 6.0
**PCO_2_(mmHg)**					
1 h	33.7 ± 4.7	30.3 ± 4.0	30.3 ± 5.3	30.2 ± 4.0	30.7 ± 3.7
4 h		30.0 ± 3.6	31.1 ± 5.3	30.0 ± 4.3	29.3 ± 6.5
12 h		31.5 ± 3.9	30.4 ± 4.5	30.0 ± 4.3	29.8 ± 6.4
24 h		32.4 ± 3.6	30.7 ± 4.7	30.1 ± 4.8	29.2 ± 5.3
**PH**					
1 h	7.37 ± 0.02	7.38 ± 0.03	7.38 ± 0.04	7.37 ± 0.03	7.36 ± 0.07
4 h		7.37 ± 0.04	7.37 ± 0.06	7.37 ± 0.03	7.36 ± 0.03
12 h		7.38 ± 0.04	7.38 ± 0.04	7.37 ± 0.03	7.39 ± 0.03
24 h		7.39 ± 0.07	7.38 ± 0.05	7.38 ± 0.05	7.38 ± 0.03
**HCO_3_^–^(mmol/L)**					
1 h	15.5 ± 2.1	15.7 ± 1.1	14.4 ± 1.3	14.0 ± 3.0	14.4 ± 2.2
4 h		15.4 ± 1.7	15.1 ± 2.4	13.8 ± 3.9	14.8 ± 2.1
12 h		16.1 ± 2.1	14.0 ± 1.7	14.9 ± 2.9	15.3 ± 2.9
24 h		14.6 ± 0.9	13.2 ± 2.2	14.2 ± 1.9	14.1 ± 1.8
**BE(mmol/L)**					
1 h	−2.4 ± 1.1	−2.1 ± 0.9	−2.0 ± 1.2	−2.1 ± 1.3	−2.3 ± 1.3
4 h		−2.1 ± 1.2	−2.1 ± 0.3	−2.6 ± 1.2	−2.7 ± 1.0
12 h		−2.3 ± 1.0	−2.1 ± 0.4	−2.4 ± 1.4	−1.9 ± 1.0
24 h		−1.7 ± 0.6	−2.3 ± 0.7	−2.4 ± 0.9	−2.1 ± 0.7
**cLac (mmol/L)**					
1 h	9.3 ± 1.9	10.7 ± 1.1	10.1 ± 1.1	10.0 ± 0.6	9.7 ± 1.8
4 h		11.6 ± 2.1*	9.5 ± 1.0^#^	9.9 ± 2.1	9.8 ± 1.8
12 h		11.6 ± 1.6*	10.1 ± 1.5	10.5 ± 1.4	9.9 ± 1.2^#^
24 h		11.5 ± 1.3*	10.5 ± 1.9	10.2 ± 1.0	10.0 ± 1.8

The data are the mean ± SD of n experiments (*n* = 8). *P < 0.05; **p < 0.01 vs. the plain control; #*p* < 0.05, ##*p* < 0.01 vs. the hypobaric chamber group.

#### Vital Organ Injury Marker

**FIGURE 3:**
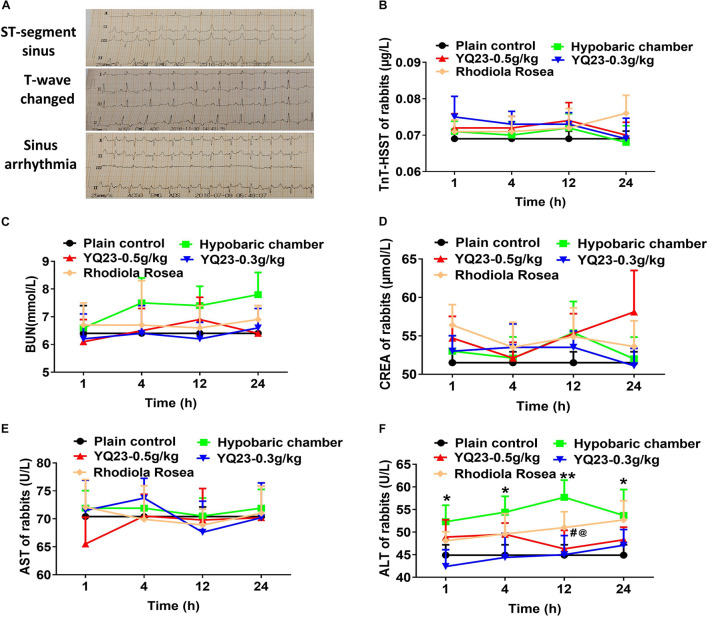
The effect of YQ23 on vital organ injury in hypobaric chamber rabbits. **(A)** The representative traces of ST-segment sinus, T-wave changed and sinus arrhythmia in hypobaric chamber rabbits (*n* = 8); **(B–D)** the benefits of YQ23 on cardiac injury **(B)**, kidney injury marker BUN **(C),** and CREA **(D)**, and liver injury marker AST **(E)** and ALT **(F)**. The data are the mean ± SD of n experiments (*n* = 8). *p < 0.05, **p < 0.01 the hypobaric chamber groups vs. the plain control; ^#^*p* < 0.05, the YQ23-0.5 g/kg groups vs. the hypobaric chamber groups; ^@^*p* < 0.05, the YQ23-0.3g/kg groups vs. the hypobaric chamber groups.

To investigate whether exposure to the hypobaric chamber induces organ injury and YQ23 alleviates the cardiac, liver, and renal injury, the ECG, cardiac injury markers, liver injury markers, and renal function were measured. In the present study, sinus arrhythmia, ST-segment depression, and T-wave changes were used to reflect the cardiomyocyte hypoxia. The results showed that the number of rabbits with sinus arrhythmia, ST-segment depression, and T-wave change increased gradually when the exposure time was prolonged in the hypobaric chamber. YQ23 treatment significantly decreased the number of rabbits with sinus arrhythmia, ST-segment depression, and T-wave change (Table 3 and Figure 3A). Furthermore, the results showed that the cardiac injury marker, TnT-HSST, as well as renal injury marker including BUN and CREA, did not markedly change after exposure to hypobaric chamber environment in rabbits. Meanwhile, YQ23 treatment did not induce an increase of these parameters (Figures 3B–D), suggesting a non-toxic effect. The blood ALT was elevated in hypobaric chamber rabbits and arrived at the peak at 12 h (44.9 ± 6.5 vs. 57.7 ± 10.7, *p* < 0.01), while AST had no change (Figures 3E,F). YQ23 treatment inhibited the increase of blood ALT (44.9 ± 6.5 vs. 42.4 ± 10.5, *p* < 0.05) (Figures 3E,F), suggesting a potential protective effect on liver injury.

**TABLE 3:** The effect of YQ23 on ECG in hypobaric chamber rabbits.

Timepoints after therapy	Groups				
	Plain control	Hypobaric chamber	YQ23-0.5 g/kg	YQ23-0.3 g/kg	*Rhodiola rosea*
**The number of sinus arrhythmia/total number**					
1 h	0/8	1/8	1/8	0/8	0/8
4 h		3/8	1/8	0/8	1/8
12 h		3/8	1/8	1/8	1/8
24 h		4/8	0/8	0/8	1/8
**The number of ST segment sinus/total number**					
1 h	0/8	2/8	1/8	0/8	1/8
4 h		3/8	1/8	1/8	0/8
12 h		3/8	1/8	2/8	4/8
24 h		5/8	2/8	1/8	2/8
**The number of T-wave change/total number**					
1 h	0/8	2/8	1/8	0/8	2/8
4 h		1/8	1/8	1/8	1/8
12 h		2/8	0/8	1/8	1/8
24 h		2/8	1/8	0/8	1/8

#### Hemodynamics, Hemorheology, and Lung and Brain Edema

The results showed exposure to a hypobaric chamber environment did not induce the changes in hemodynamics including LVSP (left ventricular systolic pressure) and ± dp/dtmax, and the hemorheology including high, middle, and low viscosity; Additionally, YQ23 treatment did not influence these parameters. These results suggest that YQ23 did not remove endodermal nitric oxide (NO) and induce coronary artery hyper-contraction to cause myocardial ischemia and hypoxia. The lung and brain edema did not occur after an explosion to a hypobaric chamber within 24 h and YQ23 did not affect the lung and brain edema.

### Part 2: The Protective Effect of YQ23 on Acute Hypoxia Injuries of Acute Mountain Sickness (AMS) in Goats

YQ23 exerted a potential protective effect on hypobaric chamber rabbits. To further clarify the benefits of YQ23 on acute hypoxia injuries of AMS, the goat model of AMS was used and made by transporting goats from the plains (Chongqing) to the plateau (Lhasa) by plane. The same parameters were observed.

#### General Behavioral Scores

**FIGURE 4:**
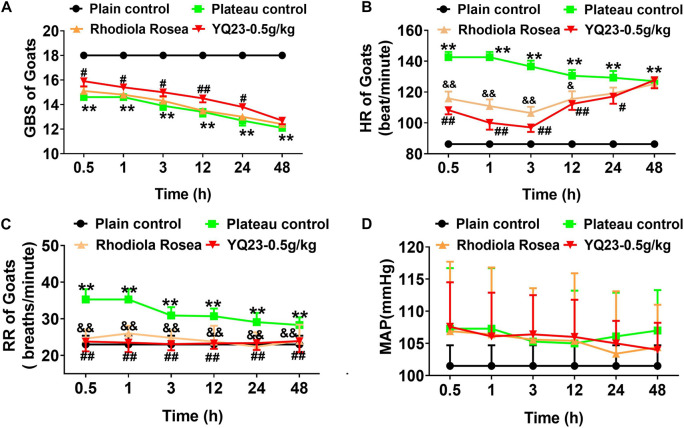
The effect of YQ23 on GBS, HR, RR, and MAP in plateau gouts. **(A)** The effect of YQ23 ON GBS; **(B,C)** The effect of YQ23 on HR and RR; **(D)** the effect of YQ23 on MAP. The data are the mean ± SD of n experiments (*n* = 8). **p < 0.01 the plateau groups vs. plain control; ^#^*p* < 0.05, ^##^*p* < 0.01 the YQ23-0.5 g/kg groups vs. the plateau groups; ^&^*p* < 0.05, ^&&^*p* < 0.01 the *R. rosea* groups vs. the plateau groups.

After the exposure to plateau hypoxia, the GBS decreased gradually (18.0 ± 0.0 vs. 13.4 ± 1.0, *p* < 0.01), showing eating less, lower muscle tension, and lower turnover. YQ23 (0.5 g/kg) treatment rapidly increased the GBS and continuously improved the ability to eat and moving for 24 h (14.5 ± 0.9 vs. 13.4 ± 1.0, *p* < 0.01), while RhRo failed to improve the GBS within 48 h (Figure 4A). Notably, the beneficial effect of YQ23 began to descend at 12 h and lost efficacy at 48 h, the reason may be the half-life of YQ23 which ranged from 12–48 h (Alayash, 2019).

#### Heart Rate, Respiratory Rate, and Mean Arterial Pressure

The HR (86.3 ± 6.2 vs. 142.7 ± 9.6, *p* < 0.01) and RR (23.0 ± 2.4 vs. 35.3 ± 2.8 *p* < 0.01) of goats increased after being transported to Lhasa. YQ23 treatment decreased the HR (100.1 ± 12.6 vs. 142.7 ± 9.6, *p* < 0.01) and RR (23.5 ± 2.6 vs. 35.3 ± 2.8, *p* < 0.01), and RhRo did the same (Figures 4B,C). The beneficial effect of YQ23 and RhRo on HR weakened gradually after 12 h (Figure 4B). The MAP did not change significantly within 48 h after arriving at Lhasa. Meanwhile, YQ23 treatment did not influence MAP (Figure 4D).

#### Blood Gases

The level of PaO_2_ (106.8 ± 7.2 vs. 67.0 ± 5.1, *p* < 0.01) and SaO_2_ (96.8 ± 1.9 vs. 72.8 ± 4.1, *p* < 0.01) markedly decreased in plateau goats after being transported to Lhasa. YQ23 treatment and RhRo treatment improved these parameters (Table 4). In addition, the level of blood lactic acid markedly increased in plateau goats (7.1 ± 0.4 vs. 10.2 ± 0.6, *p* < 0.05), YQ23 reduced the blood lactic acid in the earlier period (8.5 ± 0.4 vs. 10.2 ± 0.6, *p* < 0.05), while RhRo did not influence blood lactic acid (Table 4). There was no significant difference in the other parameters of blood gas including HCO_3_–, PaCO_2_, PH, and BE (Table 4).

**TABLE 4:** The effect of YQ23 on blood gases in goats.

Treatment time	Groups			
	Plain control	Plateau control	*Rhodiola rosea*	YQ23 0.5 g/kg
**PaO_2_ (mmHg)**				
30 min	106.8 ± 7.2		77.3 ± 5.5	80.1 ± 3.8^#^
1 h		67.0 ± 5.1	81.5 ± 5.0^#^	82.6 ± 3.2^#^
3 h		65.6 ± 4.5**	79.9 ± 5.0^#^	80.5 ± 6.0^#^
12 h		66.1 ± 5.8**	77.4 ± 6.6	78.8 ± 5.4
24 h		66.1 ± 6.7**	75.1 ± 4.5	78.9 ± 3.1
48 h		67.9 ± 5.5**	74.8 ± 5.3	76.9 ± 3.6
**SaO_2_(%)**				
30 min	96.8 ± 1.9		88.1 ± 5.1	89.8 ± 1.7
1 h		72.8 ± 4.1**	90.9 ± 3.5^#^	92.0 ± 1.9##
3 h		74.1 ± 3.2**	88.3 ± 6.6	87.6 ± 3.2
12 h		74.0 ± 4.3**	86.6 ± 4.5	90.1 ± 2.3^#^
24 h		73.1 ± 4.8**	88.0 ± 2.7	91.3 ± 1.8##
48 h		73.7 ± 3.2**	87.4 ± 3.1	90.4 ± 1.8^#^
**PaCO_2_ (mmHg)**				
30 min	35.8 ± 3.9		31.6 ± 2.9	32.1 ± 2.7
1 h		28.3 ± 3.6	30.9 ± 1.4	32.5 ± 2.1
3 h		30.6 ± 3.4	35.1 ± 3.0	36.6 ± 1.5
12 h		30.4 ± 4.2	31.6 ± 3.2	32.6 ± 2.0
24 h		29.9 ± 3.5	31.8 ± 4.1	31.5 ± 4.1
48 h		29.4 ± 2.6	32.0 ± 3.4	33.3 ± 3.2
**pH**				
30 min	7.39 ± 0.02		7.40 ± 0.06	7.40 ± 0.04
1 h		7.38 ± 0.02	7.39 ± 0.05	7.40 ± 0.04
3 h		7.40 ± 0.03	7.40 ± 0.05	7.41 ± 0.04
12 h		7.39 ± 0.03	7.38 ± 0.04	7.41 ± 0.04
24 h		7.40 ± 0.03	7.38 ± 0.05	7.41 ± 0.06
48 h		7.35 ± 0.05	7.40 ± 0.04	7.41 ± 0.05
**HCO_3_^–^ (mmol/L)**				
30 min	21.8 ± 1.9		19.5 ± 2.0	18.3 ± 3.4
1 h		18.1 ± 4.0	19.3 ± 0.7	18.6 ± 2.2
3 h		21.4 ± 4.8	20.6 ± 1.5	21.3 ± 1.8
12 h		21.1 ± 4.9	19.9 ± 3.0	18.7 ± 1.5
24 h		20.8 ± 4.3	20.0 ± 3.7	18.8 ± 3.1
48 h		20.2 ± 3.4	20.1 ± 3.1	20.4 ± 1.9
**BE (mmol/L)**				
30 min	−2.8 ± 0.9		−2.8 ± 1.6	−1.3 ± 1.5
1 h		−3.0 ± 0.9	−2.7 ± 1.2	−2.7 ± 1.4
3 h		−2.1 ± 0.7	−1.8 ± 0.9	−1.8 ± 1.3
12 h		−3.2 ± 1.0	−2.1 ± 2.0	−2.2 ± 1.1
24 h		−1.7 ± 1.0	−1.9 ± 1.1	−2.0 ± 1.0
48 h		−2.2 ± 1.0	−1.4 ± 1.1	−1.6 ± 1.0
**Lac (mmol/L)**				
30 min	7.1 ± 0.4		9.3 ± 0.7	9.0 ± 0.6
1 h		10.2 ± 0.6*	8.7 ± 0.8^#^	8.5 ± 0.4^#^
3 h		9.9 ± 0.8*	9.2 ± 0.3	9.0 ± 0.4
12 h		10.0 ± 0.3	9.3 ± 0.3	9.1 ± 0.4
24 h		9.9 ± 0.3	9.5 ± 0.5	9.2 ± 0.3
48 h		9.1 ± 0.2	9.4 ± 0.7	8.9 ± 0.6

The data are the mean ± SD of n experiments (*n* = 8). *P < 0.05; **p < 0.01 vs. the plain control; #*p* < 0.05, ##*p* < 0.01 vs. the plateau group.

#### Multiple Organ Injury Markers

**FIGURE 5:**
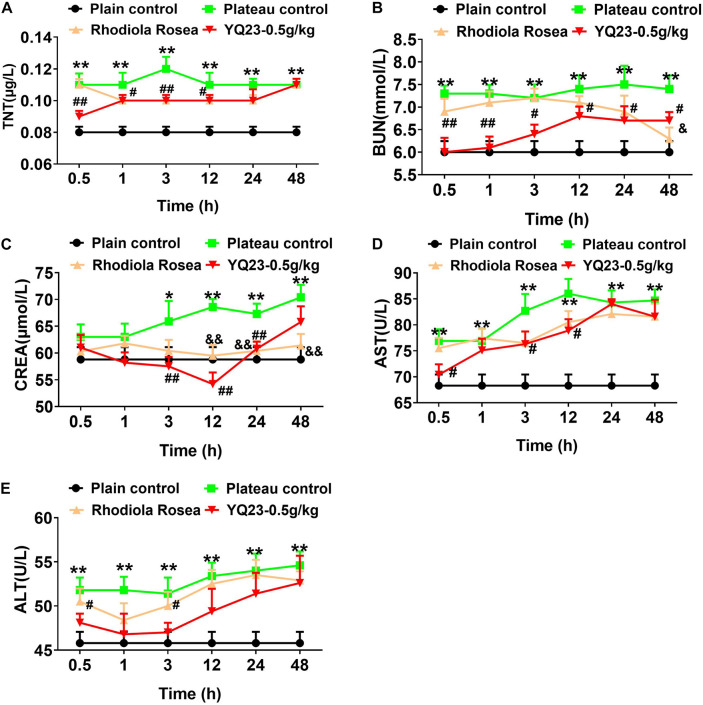
The effect of YQ23 on vital organ function in plateau gouts. **(A–E)** The benefits of YQ23 on cardiac injury **(A)**, kidney injury marker BUN **(B)** and CREA **(C)**, and liver injury marker AST **(D)** and ALT **(E)**. The data are the mean ± SD of n experiments (*n* = 8). *p < 0.05, **p < 0.01, the plateau groups vs. the plain control; ^#^*p* < 0.05, ^##^*p* < 0.01, the YQ23-0.5 g/kg groups vs. the plateau groups; ^&^*p* < 0.05, ^&&^*p* < 0.01 the *R. rosea* groups vs. the plateau groups.

Similar to the results in the hypobaric chamber rabbits, the occurrences of sinus arrhythmia, ST-segment depression, and T-wave change increased significantly in goats. Both RhRo and YQ23 decreased the incidence of sinus arrhythmia, ST-segment depression, and T-wave change (Table 5). The effect of YQ23 on sinus arrhythmia and ST-segment depression was much better than RhRo (Table 5). When compared with the results in the hypobaric chamber rabbits, the damage to the liver, kidney, and heart in plateau goats was worsening. The blood levels of TNT, BUN, CREA, AST, and ALT increased in plateau goats within 3 h (Figures 5A–E). YQ23 treatment significantly decreased the blood level of AST, ALT, TNT, BUN, and CREA (Figures 5A–E), without a non-toxic effect on organ function.

**TABLE 5:** The effect of YQ23 on ECG in goats.

Treatment time	Groups			
	Plain control	plateau control	*Rhodiola Rosea*	YQ23 0.5 g/kg
**The number of sinus arrhythmia/total number**				
30 min	0/8		1/8	1/8
1 h		2/8	2/8	1/8
3 h		2/7	2/8	1/8
12 h		2/7	2/8	2/8
24 h		2/7	2/8	2/8
48 h		3/7	2/8	1/7
**The number of ST segment sinus/total number**				
30 min	0/8		1/8	0/8
1 h		2/8	1/8	0/8
3 h		2/7	2/8	1/8
12 h		2/7	2/8	1/8
24 h		2/7	2/8	1/8
48 h		2/7	2/8	1/7
**The number of T-wave change/total number**				
30 min	0/8		2/8	1/8
1 h		2/8	1/8	1/8
3 h		2/7	1/8	1/8
12 h		2/7	1/8	1/8
24 h		3/7	1/8	1/8
48 h		3/7	1/8	2/7

#### Hemorheology, Lung, and Brain Edema

Similar to the results in the hypobaric chamber, the hemorheology did not change including high, middle, and low viscosity in plateau goats. The lung and brain edema did not occur within 48 h in plateau goats.

## Discussion

YQ23 is a novel bovine-derived, stabilized, cross-linked HBOC, which takes natural HB as its source and has the function of carrying and releasing oxygen after double aspirin crosslinking and chemical modifications. Our previous study found that YQ23 was capable of carrying and delivering oxygen and was beneficial for hemorrhagic shock and sepsis by protecting vital organ function. In addition, YQ23 rescued the barrier function of vascular endothelium by preserving the endothelial glycocalyx and intercellular junctions and improving cellular mitochondrial function (Zhao et al., 2020). This study verified the protective effect of YQ23 on acute hypoxia injuries of AMS. YQ23 treatment significantly improved the ability of general behavior, such as food intake, activity ability, and turning, and ameliorated the general vital signs including HR and RR. Furthermore, YQ23 treatment alleviated the vital organ injuries including heart, liver, and kidney, and improved the homeostasis including PaO_2_, SaO_2_, and blood lactic acid.

Acute mountain sickness is an anoxic reaction symptom that can endanger life seriously if not treated (Rolla et al., 2002). Currently, the major prevention and treatment measures for AMS included oxygen inhalation, glucocorticoid, and Acetazolamide (Tang et al., 2014; Ou et al., 2020; Toussaint et al., 2020). Oxygen inhalation is most effective for AMS. However, the heavyweight of oxygen tanks is not conducive to transport, and once the oxygen intake is stopped, its protective effect will immediately weaken, lasting for a short time. In the present study, we found that YQ23 effectively improves the symptoms of AMS. The beneficial duration can be up to at least 24 h. Compared with previous studies that found the protective duration of HBOC is generally between 7 and 17 h (Weiskopf and Silverman, 2013), this study showed the protection duration of YQ23 was found to be up to 24 h, suggesting that YQ23 has a longer protection period and has a better effect on acute hypoxia injuries of AMS at a single drug delivery.

Hypoxia is the main pathogenic factor of AMS. The decrease of oxygen saturation caused by hypoxia will lead to an increase in ventilation. However, in our results, the PaCO_2_ and HCO_3_^–^ did not change significantly. The main reason is that in our model, animals can alleviate hypoxia by compensation, without causing hyperventilation. YQ23 can further improve the symptoms of hypoxia by carrying oxygen. In addition, the results also showed that YQ23 could slow down the HR. We think this is a protective effect because, in the early stage of hypoxia, the acceleration of HR can increase cardiac output and alleviate the symptoms of hypoxia. However, long-term high HR is harmful to the heart. YQ23 can alleviate the symptoms of hypoxia by carrying oxygen, reduce the HR and protect the myocardium. Because of the adverse effect on cardiac, gastrointestinal, and renal in preclinical and clinical studies (Burhop et al., 2004; Rentsendorj et al., 2016; Weiskopf et al., 2017), the application and development of HBOC have been suspended in the last decade, although HBOCs have been proved to improve oxygen saturation and utilization. The cardiac side effect of HBOCs ascribed to scavenging of endothelial-derived NO (Alayash, 2014), which induced the hyper-constriction of the coronary arteries and subsequently lead to myocardial ischemia and hypoxia. In this study, YQ23 improved the myocardial oxygen supply and significantly decreased the incidence of sinus arrhythmia, ST-segment depression, and T-wave change without inducing myocardial ischemia and hypoxia. The cardiac injury marker TNT decreased after YQ23 treatment. Furthermore, YQ23 decreased the liver injury markers and renal injury markers.

Scavenging of endothelial-derived NO is also the major cause of the imbalance of hemodynamics for HBOCs (Risbano et al., 2015). This study showed YQ23 did not induce acute blood pressure elevation and any adverse effects on the hemodynamics including LVSP and ±dp/dtmax in plateau goats and hypobaric chamber rabbits. Although, our previous study found that YQ23 increased the MAP of sepsis rats, this effect may be associated with improved tissue oxygen supply and protection of mitochondrial function (Kuang et al., 2019) rather than scavenging endothelial-derived NO.

It is widely accepted that the nephrotoxicity of HBOC is due to the production of reactive oxygen species (ROS) including anionic superoxide radicals (O_2_^⋅^) and hydrogen peroxide (H_2_O_2_) and dimers of hemoglobin (Ascenzi et al., 2019). Our previous showed YQ23 did not induce the production of ROS and SOD (Kuang et al., 2019). Although the blood level of ROS and dimers of hemoglobin were not determined in plateau goats and hypobaric chamber rabbits in the present study, we found that YQ23 treatment significantly reduced the blood CREA and BUN in plateau goats and imposed the protective effect on kidney function, indicating most of them have stable tetramer structures rather than nephrotoxic dimer structures.

In this study, there are some different results. For instance, RhRo increased the levels of PaO_2_ and SaO_2_ in plateau goats but failed to improve the PaO_2_ and SaO_2_ in hypobaric chamber rabbits. Moreover, RhRo decreased HR in plateau goats but was incapable of reducing HR in rabbits. The inconformity may be due to the different species and systemic reactions to the RhRo.

### Limitations

There are some limitations in this study, including: (1) the hypobaric chamber rabbit and plateau goat model cannot fully simulate the AMS; (2) the mechanism of the YQ23 protective effect and the role of mitochondrial function in this process were not illuminated; (3) the biocompatibility of YQ23 and whether it induces an immune response was unclear; (4) as a blood substitute, whether YQ23 affects systematic coagulation function or peripheral resistance was not observed in this study.

## Conclusion

YQ23 has an oxygen-carrying and releasing function. YQ23 plays an important role in improving the acute hypoxia symptoms of AMS by increasing the oxygen content in the plateau blood and alleviating heart, kidney, and liver injuries. This study suggests that the novel HBOCs could be used as an oxygen-carrying/releasing drug for the symptom improvement of AMS.

## Data Availability Statement

The raw data supporting the conclusions of this article will be made available by the authors, without undue reservation.

## Ethics Statement

The animal study was reviewed and approved by the Research Council and Animal Care and Use Committee of the Research Institute of Surgery.

## Author Contributions

JZ, L-ML, and TL had full access to all of the data in the study and take responsibility for the integrity of the data and the accuracy of the data analysis. L-ML and TL contributed to the study conception and design and supervision of the studies and the obtaining of funding. YZ, YW, Q-GY, X-YP, and Q-HL contributed to the study design and execution, the data acquisition, analysis, and interpretation. BL and FT contributed to the quality control of YQ23. TL contributed to the study conception and drafting of the manuscript. All the authors read and approved the final manuscript.

## Conflict of Interest

BL and FT were employed by the company New Beta Innovation Limited. The remaining authors declare that the research was conducted in the absence of any commercial or financial relationships that could be construed as a potential conflict of interest.

## Publisher’s Note

All claims expressed in this article are solely those of the authors and do not necessarily represent those of their affiliated organizations, or those of the publisher, the editors and the reviewers. Any product that may be evaluated in this article, or claim that may be made by its manufacturer, is not guaranteed or endorsed by the publisher.
