# Supplementary material for: The Protective Effect of a Novel Cross-Linked Hemoglobin-Based Oxygen Carrier on Hypoxia Injury of Acute Mountain Sickness in Rabbits and Goats
Source: Front Physiol. 2021 Sep 27;12:690190. doi: 10.3389/fphys.2021.690190 (PMC8502813; doi:10.3389/fphys.2021.690190)
Supplement: Supplementary file 1 [file Table_6.DOCX]

| **Supplemental Table variables that effect the General Behavioral Scores (GBS)** | | |
| --- | --- | --- |
| Order | GBS Index | Variables effecting GBS |
| 1 | Feeding behavior | Environmental oxygen content |
|  |  | Environmental temperature |
| 2 | Righting score | Heart rate |
|  |  | Respiratory rate |
|  |  | Environmental oxygen content |
|  |  | Blood pressure |
| 3 | Muscle tone | Heart rate |
|  |  | Respiratory rate |
|  |  | Environmental oxygen content |
|  |  | Blood pressure |
| 4 | Motor ability | Heart rate |
|  |  | Respiratory rate |
|  |  | Environmental oxygen content |
|  |  | Environmental temperature |
| 5 | Olfactory stimuli | Environmental oxygen content |
|  |  |  |
| 6 | Grasping reaction | Heart rate |
|  |  | Respiratory rate |
|  |  | Blood pressure |
